# Statistical evidence for high‐penetrance MODY‐causing genes in a large population‐based cohort

**DOI:** 10.1002/edm2.372

**Published:** 2022-10-08

**Authors:** Liana K. Billings, Zhuqing Shi, W. Kyle Resurreccion, Chi‐Hsiung Wang, Jun Wei, Toni I. Pollin, Miriam S. Udler, Jianfeng Xu

**Affiliations:** ^1^ Department of Medicine NorthShore University HealthSystem Skokie Illinois USA; ^2^ University of Chicago Pritzker School of Medicine Chicago Illinois USA; ^3^ Program for Personalized Cancer Care NorthShore University HealthSystem Evanston Illinois USA; ^4^ Department of Medicine, Division of Endocrinology, Diabetes and Nutrition, and Program in Personalized and Genomic Medicine University of Maryland School of Medicine Baltimore Maryland USA; ^5^ Diabetes Unit Massachusetts General Hospital Boston Massachusetts USA; ^6^ Department of Medicine Harvard Medical School Boston Massachusetts USA

**Keywords:** diabetes genetics, maturity‐onset diabetes of the young, MODY, pathogenic variant, variant curation

## Abstract

**Aims:**

Numerous genes have been proposed as causal for maturity‐onset diabetes of the young (MODY). Scoring systems to annotate mutation pathogenicity have been widely used; however, statistical evidence for being a highly penetrant MODY gene has not been well‐established.

**Methods:**

Participants were from the UK Biobank with whole‐exome sequencing data, including 14,622 with and 185,509 without diagnosis of diabetes. Pathogenic/likely pathogenic (P/LP) mutations in 14 reported and 3 possible MODY genes were annotated using American College of Medical Genetics criteria. Evidence for being a high‐penetrant MODY gene used two statistical criteria: frequency of aggregate P/LP mutations in each gene are (1) significantly more common in participants with a diagnosis of diabetes than without using the SKAT‐O (*p* < .05) and (2) lower than the maximum credible frequency in the general population.

**Results:**

Among the 17 genes, 6 (*GCK*, *HNF1A*, *HNF4A*, *NEUROD1*, *KCNJ11* and *HNF1B*) met both criteria, 7 (*ABCC8, KLF11, RFX6, PCBD1, WFS1, INS and PDX1*) met only one criterion, and the remaining 4 (*CEL, BLK*, *APPL1* and *PAX4*) failed both criteria, and were classified as ‘consistent’, ‘inconclusive’ and ‘inconsistent’ for being highly penetrant diabetes genes, respectively. Diabetes participants with mutations in the ‘consistent’ genes had clinical presentations that were most consistent with MODY. In contrast, the ‘inconclusive’ and ‘inconsistent’ genes did not differ clinically from non‐carriers in diabetes‐related characteristics.

**Conclusions:**

Data from a large population‐based study provided novel statistical evidence to identify 6 MODY genes as consistent with being highly penetrant. These results have potential implications for interpreting genetic testing results and clinical diagnosis of MODY.

## Graphical abstract

Study design and results for assessing statistical evidence of candidate MODY‐causing genes being high‐penetrance in the UK Biobank, a large population‐based cohort.

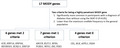

## 1 INTRODUCTION

Maturity‐onset diabetes of the young (MODY) is the most common type of monogenic diabetes.^1^ While MODY is classically characterized by early‐onset diabetes (<25 years), insulin independence and autosomal dominant inheritance, it is a genetically and clinically heterogeneous group of diabetes resulting from mutations in genes mostly involved in pancreatic beta cell function.^1^ MODY can be difficult to diagnose clinically due to overlapping features with type 1 and type 2 diabetes. A correct clinical diagnosis of MODY requires genetic testing of MODY‐causing genes. Ultimately, the genetic diagnosis can direct the therapeutic intervention for diabetes treatment.

Implicating MODY‐causing genes is complicated by several other confounding factors, including poor statistical power due to limited number of patients and pedigrees, uncertainty in classifying mutation pathogenicity, and relevance of in vitro functional analysis. To date, 14 disease‐causing genes have been reported for distinct subtypes of MODY.^2^ Recently, three additional candidate genes (*WFS1*, *RFX6* and *PCBD1*) for MODY‐like phenotypes were proposed.^3^, ^4^, ^5^ The vast majority of these genes were identified through genetic linkage analyses in a limited number of families with atypical diabetes (OMIM #606391). While statistical evidence for being high‐penetrant MODY genes has been well‐established for some, it is weak or inconsistent for others.^6^

The objective of the current study is to systematically evaluate statistical evidence of proposed MODY genes for being highly penetrant. Leveraging a large population‐based cohort, we applied a novel and indirect approach to assess the statistical evidence by estimating mutation carrier rates of genes in participants with or without a diagnosis of diabetes, rather than diagnosis of MODY per se. This approach overcomes the major challenge of accurately defining MODY clinically and is based on a simple and justifiable assumption that a higher proportion of people with MODY exists in participants with than without a diagnosis of diabetes. Under this assumption, aggregate pathogenic and likely pathogenic (P/LP) mutations in high‐penetrant MODY‐causing genes are expected to meet two statistical criteria: (1) significantly more common in participants with than without a diagnosis of diabetes and (2) rarely observed in participants from the general population.

## 2 METHODS

Study participants were from the UK Biobank (UKB), a large population‐based cohort with extensive genetic and phenotypic data available for approximately 500,000 individuals from across the United Kingdom aged between 40 and 69 at recruitment (accessed under Application Number: 50295).^7^ Diabetes diagnosis International Classification of Diseases‐10 codes in medical records (E10 for type 1 diabetes, E11 for type 2 diabetes, E12 for other specified diabetes and E14 for unspecified diabetes) as well as self‐report.

Whole‐exome sequencing (WES) data were available for ~40% of the UKB participants at the time of this analysis. The 14 commonly proposed MODY genes (*HNF4A*, *GCK*, *HNF1A*, *PDX1*, *HNF1B*, *NEUROD1*, *KLF11*, *CEL*, *PAX4*, *INS*, *BLK*, *ABCC8*, *KCNJ11* and APPL1),^2^ and three newly proposed candidate MODY genes (*WFS1*, *RFX6* and *PCBD1*) were evaluated.^3^, ^4^, ^5^ We first annotated variants using a pipeline based on the American College of Medical Genetics (ACMG) criteria and identified 591 potential P/LP variants.^8^ These variants were further annotated manually based on the sequence variant interpretation (SVI) guidelines.^8^, ^9^ The detailed criteria for each variant are listed in Table S1. A total of 102 variants were considered as P/LP.

The transcript expression‐aware annotation was examining proportion expressed across transcripts (pext) scores of exons in these genes in the pancreas (Table S1).^10^

Evidence for high‐penetrant MODY genes was indirectly assessed using two statistical criteria: aggregation of P/LP mutations in a gene is (1) significantly more common in participants with than without a diagnosis of diabetes and (2) rarely observed in participants from the UKB.

For the first criterion, a gene‐based association test was performed using the SKAT‐O as well as Fisher's exact test. SKAT‐O is a unified approach that optimally combines the burden test and the nonburden sequence kernel association test (SKAT).^11^ Burden test is more powerful when most mutations in a gene are causal, whereas SKAT is more powerful when a large fraction of the mutations in a gene are noncausal or the effects of causal variants are in different directions. SKAT‐O association test was adjusted for age, gender and genetic background (the top 10 principal components provided by the UKB). Odds ratio (OR) and 95% confidence interval (CI) were derived from the Fisher exact test. This gene‐based criterion is equivalent to the variant‐based pathogenic strong (PS4) criterion for P/LP mutations.

For the second criterion, the maximum credible aggregated P/LP mutation frequency in the general population was estimated for each MODY gene using the tool (http://cardiodb.org/allelefrequencyapp/) based on the following parameters: MODY prevalence (1/1000), contribution of each gene to MODY and penetrance (50%) (Table S1).^9^, ^12^ This gene‐based criterion is equivalent to the variant‐based BA1 criterion for benign variants.

Genes were then classified as ‘consistent’, ‘inconclusive’ or ‘inconsistent’ with being highly penetrant variants. Because MODY can present with atypical diabetes or overlapping clinical characteristics of type 1 and type 2 diabetes, we examined the prevalence of the gene variants by six different study‐specific diabetes phenotypes defined as classic type 1 diabetes included participants with clinical diagnosis code (ICD‐10) type 1 but not type 2 diabetes, insulin‐use, no oral anti‐hyperglycemic agents and onset of diabetes ≤20. Classic type 2 diabetes included participants with ICD‐10 type 2 but not type 1 diabetes, non‐insulin using, and onset ≥35. Non‐classic type 1 or non‐classic type 2 were the subjects who had either type 1 diabetes or type 2 diabetes only diagnosis, respectively, but did not fulfil the other classic criteria. Type 1 diabetes and type 2 diagnosis were subjects with both diagnoses. Other diabetes subjects were and diabetes diagnosis, but not defined as type 1 or type 2 diabetes. This group incorporates people diagnosed with other specified diabetes (ICD‐10 codes E12‐E14), which could include people with MODY.

Subsequently, we examined clinical characteristics of mutation carriers with and without diabetes including demographic and clinical measurements.

## 3 RESULTS

Among the 17 genes, 6 (*GCK*, *HNF1A*, *HNF4A*, *KCNJ11, NEUROD1* and *HNF1B*) met both criteria. These were classified as ‘consistent’ (Table 1). Seven genes (*ABCC8, KLF11*, *RFX6*, *PCBD1*, *WFS1, INS and PDX1*) met only one criterion and were classified as ‘inconclusive’; the first 6 genes only met criterion 1 (*p* < .05, SKAT‐O) while the last gene only met criterion 2. The remaining 4 genes (*PAX4, CEL, BLK* and *APPL1*) failed both criteria and were classified as ‘inconsistent’. *PDX1* had no P/LP mutations identified in the UKB cohort.

**TABLE 1:** Mutation carrier rates of known MODY genes in participants with and without a diagnosis of diabetes in the UK Biobank

Group	Type of MODY	Criterion 1					Criterion 2	
		No. (%) of participants		SKAT‐O *p*‐value^a^	Fisher's exact test		Aggregate allele frequency in UKB	Maximum credible population allele frequency
		Any diabetes (*N* = 14,622)	Non diabetes (*N* = 185,952)		OR (95% CI)	*p*‐value		
‘Consistent’ with being high penetrant for diabetes (met both statistical criteria)								
*GCK*	MODY 2	24 (0.164%)	8 (0.004%)	6.26E−30	38.2 (16.6–98.34)	2.94E−21	0.008%	0.040%
*HNF4A*	MODY 1	4 (0.027%)	5 (0.003%)	4.09E−10	10.18 (2.02–47.28)	.00264	0.002%	0.007%
*HNF1A*	MODY 3	12 (0.082%)	7 (0.004%)	5.92E−07	21.82 (7.92–65.41)	6.95E−10	0.005%	0.045%
*NEUROD1*	MODY 6	2 (0.014%)	0 (0%)	2.17E−05	Inf (2.39−Inf)	.00531	0.0005%	0.001%
*KCNJ11*	MODY 13	2 (0.014%)	1 (0.001%)	1.42E−06	25.43 (1.32–1484.18)	.01517	0.001%	0.001%
*HNF1B*	MODY 5	1 (0.007%)	2 (0.001%)	.04	6.36 (0.11–122.21)	.20315	0.001%	0.005%
‘Inconclusive’ with being high penetrant for diabetes (met one of two statistical criteria)								
*ABCC8*	MODY 12	2 (0.014%)	3 (0.002%)	1.64E−06	8.48 (0.71–74.02)	.04581	0.0012%	0.001%
*KLF11*	MODY 7	1 (0.007%)	4 (0.002%)	1.30E−04	3.18 (0.06–32.12)	.3151	0.0012%	0.001%
*RFX6*	New	2 (0.014%)	11 (0.006%)	.01	2.31 (0.25–10.6)	.24409	0.003%	0.001%
*PCBD1*	New	2 (0.014%)	4 (0.002%)	.01	6.36 (0.58–44.43)	.06544	0.001%	0.001%
*WFS1*	New	3 (0.021%)	15 (0.008%)	.02	2.54 (0.47–8.99)	.1394	0.004%	0.001%
*INS*	MODY 10	0 (0%)	1 (0.001%)	.70	0 (0–492.17)	1	0.0002%	0.001%
*PDX1*	MODY 4	0 (0%)	0 (0%)	\	\	\	0%	0.001%
‘Inconsistent’ with being high penetrant for diabetes (failed both statistical criteria)								
*PAX4*	MODY 9	1 (0.007%)	6 (0.003%)	.58	2.12 (0.05–17.47)	.41132	0.0017%	0.001%
*CEL*	MODY 8	0 (0%)	8 (0.004%)	.95	0 (0–7.45)	1	0.002%	0.001%
*BLK*	MODY 11	0 (0%)	16 (0.009%)	>.99	0 (0–3.3)	.62523	0.0040%	0.001%
*APPL1*	MODY 14	0 (0%)	18 (0.01%)	>.99	0 (0–2.89)	\	0.0045%	0.001%

Abbreviations: Inf, infinity; OR (95% CI), odds ratio (95% confidence interval).

^a^ Adjusted for age at recruitment, gender and genetic background.

The risk of diabetes versus non‐diabetes among carriers of P/LP in ‘consistent’ genes was considerably higher, with OR estimates ranging from 6.36 to infinity (Table 1). In contrast, ‘inconclusive’ genes demonstrated medium risk, with OR estimates ranging from 0 to 8.48. In particular, 4 genes (*INS*, *CEL, BLK* and *APPL1*) had OR estimates <1, although not statistically significant (*p* > .05). In order to address the possibility of diabetes underdiagnosis, we found no difference in grouping of the MODY candidate genes when using HbA1c > 6.5% versus <6.5% in place of the diabetes or non‐diabetes diagnosis (Table S1).

When inspecting mutation carriers among different types of diabetes phenotypes, carriers were found primarily in those with a type 2 diabetes diagnosis (classic type 2 diabetes or non‐classic type 2 diabetes), followed by other diabetes, and rarely in participants with type 1 diabetes diagnosis (Table 2). The different mutation carrier rates between participants with type 2 diabetes versus other forms of diabetes, however, were not statistically significant (*p* > .05, data not shown). These observations were found across all three gene categories.

**TABLE 2:** Mutation carrier rates of MODY genes in participants with various diabetes phenotypes in the UK Biobank

Group	Type of MODY	No. (%) of participants					
		Type 1 diabetes (T1D)		Type 2 diabetes (T2D)		Other	
		Classic (*N* = 192)	Non‐classic (*N* = 340)	Classic (*N* = 5971)	Non‐classic (*N* = 4770)	T1D and T2D diagnoses (*N* = 871)	Other diabetes (*N* = 2478)
‘Consistent’ with being high penetrant for diabetes (met both statistical criteria)							
*GCK*	MODY 2	0 (0%)	0 (0%)	16 (0.268%)	5 (0.105%)	0 (0%)	3 (0.121%)
*HNF4A*	MODY 1	0 (0%)	0 (0%)	1 (0.017%)	2 (0.042%)	1 (0.115%)	0 (0%)
*HNF1A*	MODY 3	0 (0%)	1 (0.294%)	3 (0.05%)	4 (0.084%)	1 (0.115%)	3 (0.121%)
*NEUROD1*	MODY 6	0 (0%)	0 (0%)	0 (0%)	1 (0.021%)	1 (0.115%)	0 (0%)
*KCNJ11*	MODY 13	0 (0%)	0 (0%)	1 (0.017%)	0 (0%)	1 (0.115%)	0 (0%)
*HNF1B*	MODY 5	0 (0%)	0 (0%)	0 (0%)	1 (0.021%)	0 (0%)	0 (0%)
*All 6 genes*		0 (0%)	1 (0.294%)	21 (0.352%)	13 (0.273%)	4 (0.459%)	6 (0.242%)
‘Inconclusive’ with being high penetrant for diabetes (met one of two statistical criteria)							
*ABCC8*	MODY 12	0 (0%)	0 (0%)	0 (0%)	2 (0.042%)	0 (0%)	0 (0%)
*KLF11*	MODY 7	0 (0%)	0 (0%)	0 (0%)	1 (0.021%)	0 (0%)	0 (0%)
*RFX6*	New	0 (0%)	0 (0%)	2 (0.033%)	0 (0%)	0 (0%)	0 (0%)
*PCBD1*	New	0 (0%)	0 (0%)	2 (0.033%)	0 (0%)	0 (0%)	0 (0%)
*WFS1*	New	0 (0%)	0 (0%)	1 (0.017%)	0 (0%)	1 (0.115%)	1 (0.04%)
*INS*	MODY 10	0 (0%)	0 (0%)	0 (0%)	0 (0%)	0 (0%)	0 (0%)
*PDX1*	MODY 4	0 (0%)	0 (0%)	0 (0%)	0 (0%)	0 (0%)	0 (0%)
*All 7 genes*		0 (0%)	0 (0%)	5 (0.084%)	3 (0.063%)	1 (0.115%)	1 (0.04%)
‘Inconsistent’ with being high penetrant for diabetes (failed both statistical criteria)							
*PAX4*	MODY 9	0 (0%)	0 (0%)	1 (0.017%)	0 (0%)	0 (0%)	0 (0%)
*CEL*	MODY 8	0 (0%)	0 (0%)	0 (0%)	0 (0%)	0 (0%)	0 (0%)
*BLK*	MODY 11	0 (0%)	0 (0%)	0 (0%)	0 (0%)	0 (0%)	0 (0%)
*APPL1*	MODY 14	0 (0%)	0 (0%)	0 (0%)	0 (0%)	0 (0%)	0 (0%)
All 4 genes		0 (0%)	0 (0%)	1 (0.017%)	0 (0%)	0 (0%)	0 (0%)

We also examined key diabetes‐related characteristics between carriers and non‐carriers of P/LP mutation in each of the 17 genes among the three categories of diabetes participants (Table 3). Compared to diabetes participants without any mutations in the 6 ‘consistent’ genes, mutation carriers had significantly lower age at diagnosis (*p* < .05), higher proportion of family history (*p* < .001), and lower BMI (*p* < .001), higher HDL (*p* < .001) and lower TG (*p* < .001) consistent with the key features of MODY. The participants with ‘inconclusive’ mutations did not show features consistent with MODY. There were too few participants with diabetes in ‘inconsistent’ category to adequately examine these characteristics.

**TABLE 3:** Key characteristics of participants with diabetes diagnosis by mutation carrier status in the UK Biobank

Group	Type of MODY	No. of participants	Age of diagnosis Median (IQR), (year)	No. of men (%)	No. of participants with positive FH (%)	BMI in kg/m^2^ Median (IQR)	HbA1C in mmol/mol, % Median (IQR)	HDL in mmol/L Median (IQR)	TG in mmol/L Median (IQR)
Non‐carriers in any of 17 genes		14,566	54 (46.9–60)	8734 (60%)	6258 (44.5%)	30.52 (27.27–34.43)	47 (41–56) 6.4 (5.9–7.3)	1.17 (0.99–1.38)	1.88 (1.31–2.7)
‘Consistent’ with being high penetrant for diabetes (met both statistical criteria)									
*GCK*	MODY 2	24	55 (52–60)	9 (37.5%)	13 (59.1%)	25.15 (23.39–27.74)	48 (45–50) 6.5 (6.2–6.7)	1.58 (0.79–1.55)	0.99 (0.83–1.65)
*HNF4A*	MODY 1	4	37 (33.5–43.5)	1 (25%)	4 (100%)	26.23 (23.89–27.14)	58 (52–69) 7.5 (7.0–8.4)	1.19 (−1.68 to 4.52)	2.21 (1.44–2.83)
*HNF1A*	MODY 3	12	27 (19.5–48.5)	4 (33.3%)	7 (58.3%)	27.52 (25.4–31.2)	53 (50–57) 7.0 (6.7–7.3)	1.46 (0.24–2.35)	1.12 (0.95–1.61)
*NEUROD1*	MODY 6	2	48.5 (43.75–53.25)	1 (50%)	2 (100%)	29.62 (26.21–33.03)	52 (47–57) 6.9 (6.5–7.3)	1.72 (−)	1.23 (0.99–1.48)
*KCNJ11*	MODY 13	2	49.5 (44.75–54.25)	0 (0%)	2 (100%)	32.57 (29.44–35.7)	48 (47–50) 6.5 (6.4–6.7)	1.48 (−)	1.45 (1.27–1.62)
*HNF1B*	MODY 5	1	50 (−)	0 (0%)	0 (0%)	30.99 (−)	55 (−) 7.2 (−)	1.08 (−)	1.85 (−)
All 6 genes^1^		45	52 (40.25–58.75)^c^	16 (35.6%)^d^	28 (65.1%)^b^	26.28 (23.94–30.28)^d^	50 (46–53) 6.7 (6.4–7.1)^a^	1.48 (0.92–1.63)^d^	1.12 (0.89–1.8)^d^
‘Inconclusive’ with being high penetrant for diabetes (met one of two statistical criteria)									
*ABCC8*	MODY 12	2	NA (NA‐NA)	1 (50%)	1 (50%)	40.49 (37.15–43.84)	55 (53–57) 7.2 (7–7.3)	1.25 (−)	0.93 (0.93–0.93)
*KLF11*	MODY 7	1	NA (−)	0 (0%)	1 (100%)	32.43 (−)	38 (−) 5.6 (−)	0.96 (−)	3.11 (−)
*RFX6*	New	2	51.5 (50.75–52.25)	0 (0%)	1 (50%)	30.64 (28.43–32.84)	55 (48–61) 7.1 (6.6–7.7)	1.64 (−)	1.94 (1.49–2.4)
*PCBD1*	New	2	57 (53.5–60.5)	1 (50%)	2 (100%)	33 (29.84–36.16)	73 (64–81) 8.8 (8.0–9.6)	1.18 (−)	2.98 (2.74–3.22)
*WFS1*	New	3	62 (56–64)	1 (33.3%)	2 (66.7%)	33.12 (31.19–34.26)	48 (48–53) 6.6 (6.5–7.0)	1.11 (−0.46–4.01)	1.93 (1.78–2.89)
*INS*	MODY 10	0	\		\	\	\	\	\
*PDX1*	MODY 4	0	\		\	\	\	\	\
All 7 genes^1^		10	53 (50–63)^a^	6 (60%)^a^	7 (70%)^a^	33.46 (30.05–35.32)^a^	54 (47–58) 7.0 (6.5–7.5)^a^	1.2 (0.58–1.9)^a^	2.5 (1.62–3.11)^a^
‘Inconsistent’ with being high penetrant for diabetes (failed both statistical criteria)									
*PAX4*	MODY 9	1	56 (−)	0 (0%)	1 (100%)	20.07 (−)	52 (−) 6.9 (−)	1.54 (−)	1.54 (−)
*CEL*	MODY 8	0	\	\	\	\	\	\	\
*BLK*	MODY 11	0	\	\	\	\	\	\	\
*APPL1*	MODY 14	0	\	\	\	\	\	\	\
All 4 genes^1^		1	56 (−)	0 (0%)^a^	1 (100%)^a^	20.07 (−)	52 (−) 6.9 (−)	1.54 (−)	1.54 (−)

Abbreviations: FH, family history; IQR, interquartile range; NA, not applicable; TG, triglycerides.

^1^ Adjusted (1) for age and genetic background for no. of men, and (2) for age, gender and genetic background for the remaining variables.

^a,b,c,d^ *p*‐values are comparing total carriers in each category to non‐carriers. ^a^ *p* > .05, ^b^ *p* < .05, ^c^ *p* < .01 and ^d^ *p* < .001.

Among participants without a diagnosis of diabetes (Table 4), mutation carriers with ‘consistent’ genes had significantly higher proportion of family history (*p* < .001) and higher HbA1C (*p* < .001) compared to participants without any mutations in the 17 genes. No significant differences were found in these variables for mutation carriers of ‘inconclusive’ and ‘inconsistent’ genes. Of note, the mean age at enrolment among diabetes and non‐diabetes participants was 60 (95% CI 59.88–60.12) and 56.72 (56.68–56.76), respectively (*p* < 2.2e−16). Given that MODY diabetes tends to present under the age of 35, we think it is low likelihood that the patient may not have developed diabetes by the age of enrolment in the study. We examined additional clinical variables which are included in Table S1.

**TABLE 4:** Key characteristics of subjects without diabetes diagnosis by mutation carrier status in the UK Biobank

Group	Type of MODY	No. of participants	Age at recruitment Median (IQR), (year)	No. of men (%)	No. of participants with positive FH (%)	BMI in kg/m^2^ Median (IQR)	HbA1C in mmol/mol, % Median (IQR)	HDL in mmol/L Median (IQR)	TG in mmol/L Median (IQR)
Non‐carriers in any of 17 genes		185,843	57.5 (50.5–63.5)	81,301 (43.7%)	38,795 (21.3%)	26.47 (23.97–29.45)	35 (33–37) 5.34 (5.1–5.6)	1.43 (1.2–1.7)	1.45 (1.03–2.09)
‘Consistent’ with being high penetrant for diabetes (met both statistical criteria)									
*GCK*	MODY 2	8	58.5 (49.5–60.5)	2 (25%)	5 (62.5%)	25.28 (22.28–28.09)	48.15 (45–50) 6.55 (6.3–6.7)	1.55 (1.28–1.59)	1.05 (0.87–1.11)
*HNF4A*	MODY 1	5	56.5 (55.5–66.5)	2 (40%)	2 (40%)	26.19 (24.35–27.29)	39. (37–40) 5.73 (5.5–5.8)	1.56 (1.34–1.74)	1.42 (0.56–1.68)
*HNF1A*	MODY 3	7	62.5 (58–63)	3 (42.9%)	3 (42.9%)	28.4 (25.46–30.02)	35.4 (33–43) 5.38 (5.2–6.1)	1.54 (1.49–1.74)	1.57 (1.15–1.86)
*NEUROD1*	MODY 6	0	\	\	\	\	\	\	\
*KCNJ11*	MODY 13	1	62.5 (–)	0 (0%)	1 (100%)	29.31 (−)	41.4 (−) 5.93 (−)	1.16 (−)	1.93 (−)
*HNF1B*	MODY 5	2	53.5 (50.5–56.5)	1 (50%)	2 (100%)	24.29 (21.36–27.21)	36.75 (35.62–37.88) 5.5 (5.4–5.61)	1.99 (1.99–1.99)	2.5 (1.74–3.25)
All 6 genes^1^		23	59.5 (54.5–62.5)a	9 (39.1%)^a^	13 (65%)^d^	27.11 (23.26–29.06)^a^	41.05 (36.1–45.8) 5.9 (5.44–6.33)^b^	1.55 (1.42–1.74)^a^	1.18 (0.92–1.77)^a^
‘Inconclusive’ with being high penetrant for diabetes (met one of two statistical criteria)									
*ABCC8*	MODY 12	3	46.5 (46.5–47.5)	0 (0%)	0 (0%)	27.21 (27.17–27.87)	36.4 (31.95–38) 5.47 (5.06–5.62)	1.43 (1.14–1.69)	1.49 (1.13–1.82)
*KLF11*	MODY 7	4	47.5 (42.25–53.75)	0 (0%)	0 (0%)	28.56 (25.11–32.07)	35.5 (34.62–35.8) 5.39 (5.31–5.42)	1.49 (1.32–1.74)	1.49 (1.34–1.7)
*RFX6*	New	11	61.5 (57–66)	4 (36.4%)	5 (45.5%)	27.14 (25.07–29.98)	37.6 (34.2–39.55) 5.6 (5.27–5.76)	1.44 (1.26–1.55)	1.47 (0.94–2.71)
*PCBD1*	New	4	53.5 (52.5–56.5)	1 (25%)	2 (50%)	26.11 (24.41–27.65)	37.05 (36.52–39) 5.53 (5.48–5.71)	1.22 (1.11–1.61)	0.92 (0.86–0.98)
*WFS1*	New	15	56.5 (48–60.5)	6 (40%)	2 (13.3%)	28.37 (24.99–29.44)	34.1 (32.25–35.55) 5.26 (5.09–5.39)	1.17 (1.09–1.35)	2.27 (1.45–2.59)
*INS*	MODY 10	1	65.5 (–)	0 (0%)	0 (0%)	24.79 (−)	39 (−) 5.71 (−)	1.86 (−)	0.68 (−)
*PDX1*	MODY 4	0	\	\		\	\	\	\
All 7 genes^1^		38	56.5 (49–61.5)^a^	12 (31.6%)^a^	9 (23.7%)^a^	27.17 (24.96–29.95)^a^	35 (33–38) 5.4 (5.2–5.6)^a^	1.38 (1.17–1.62)^a^	1.47 (0.93–2.3)^a^
‘Inconsistent’ with being high penetrant for diabetes (failed both statistical criteria)									
*PAX4*	MODY 9	6	57.5 (46.5–64.75)	2 (33.3%)	2 (33.3%)	27.43 (22.98–28.54)	34.1 (32.3–36.12) 5.26 (5.1–5.45)	1.35 (1.21–1.55)	2.87 (1.88–3.58)
*CEL*	MODY 8	8	61.5 (46.75–63)	3 (37.5%)	0 (0%)	27.38 (26.35–28.21)	36.25 (34.92–37.45) 5.46 (5.34–5.57)	1.48 (1.17–1.55)	1.17 (0.97–1.56)
*BLK*	MODY 11	16	56.5 (51.5–61.75)	6 (37.5%)	3 (18.8%)	24.47 (22.1–28.64)	35.4 (32.9–37.25) 5.38 (5.15–5.55)	1.35 (1.2–1.55)	1.4 (1.07–1.71)
*APPL1*	MODY 14	18	58.5 (53–62.25)	7 (38.9%)	4 (22.2%)	26.7 (23.75–29.98)	35.25 (33.7–37.48) 5.37 (5.22–5.57)	1.36 (1.17–1.76)	1.9 (1.21–2.68)
All 4 genes^1^		48	58.5 (48.5–62.5)^a^	18 (37.5%)^a^	9 (18.8%)^a^	26.62 (23.23–28.52)^a^	35.4 (33.4–37.35) 5.38 (5.2–5.56)^a^	1.36 (1.2–1.57)^a^	1.51 (1.08–2)^a^

Abbreviations: FH, family history; IQR, interquartile range; TG, triglycerides.

^1^ Adjusted (1) for age and genetic background for no. of men, and (2) for age, gender and genetic background for the remaining variables.

^a,b^ *p*‐values are comparing total carriers in each category to non‐carriers. ^a^ *p* > .05 and ^b^ *p* < .001.

Finally, we found that the vast majority of annotated P/LP mutations were in transcripts highly expressed in pancreatic tissue, with pext scores >0.4 (Table S1). However, mutations in several genes were at exons with low pext scores, including a score of 0 for all mutations in *BLK* and *PAX4* (i.e. not expressed in pancreatic tissue), scores of 0.18–0.27 for *ABCC8* and a score of 0.24 for exon 1 of *KLF11*. If applying the transcript expression‐aware annotation method, no mutations in *BLK*, *PAX4* and *ABCC8* would be classified as P/LP. Annotation of *KLF11* was not affected.

## 4 DISCUSSION

Leveraging the genetic data of over 200,000 participants with and without a diagnosis of diabetes in a population‐based cohort, we were able to systematically evaluate statistical evidence of known or suspected MODY genes for being highly penetrant. Two statistical criteria were used to assess highly penetrant MODY‐causing genes: their aggregated P/LP variants are (1) significantly more common in participants with than without a diagnosis of diabetes and (2) rarely observed in participants from the general population. Among the 17 known MODY genes, 6 met both criteria, ‘consistent’, 7 met only one criterion, ‘inconclusive’, and the remaining 4 failed both criteria, ‘inconsistent’. These results provide an important piece of genetic evidence for evaluating gene‐disease clinical validity by Clinical Genome Resource (ClinGen).^13^

Most previous studies of MODY genes rely on detailed clinical evaluation of diabetes participants and their relatives.^3^, ^4^, ^5^, ^9^ A major challenge of these studies is the reliability of MODY diagnosis, which is complicated by the considerable overlap of clinical presentations between MODY, type 1 diabetes and type 2 diabetes.^1^ The alternative and indirect approach used in this study bypassed the need for defining MODY clinical diagnosis. The large number of study participants in the UKB, especially those without a diagnosis of diabetes, makes this approach feasible. The validity of this approach is supported by its simple and justifiable assumption as well as the successful confirmation of 6 well‐established MODY genes. The statistical power of this indirect approach, however, relies on the proportion of MODY in participants with or without a diagnosis of diabetes. Considering that MODY likely accounts for a small proportion of diabetes (0.4%),^1^ a large sample size such as the UKB is necessary to obtain the statistical power to detect the small signals from MODY‐causing genes.

While both statistical criteria are important to assess the validity of high‐penetrant MODY genes, a critical aspect of our study is the ability to estimate mutation carrier rates in a population‐based cohort. A higher mutation carrier rate in the general population and unaffected participants is strong evidence against being a high‐penetrant gene. For example, the mutation carrier rate of *WFS1* was significantly higher in cases than controls in the UKB; OR = 1.85, *p* = .02. However, its considerably high aggregate P/LP mutation allele frequency in the UKB (0.016%), 16‐times higher than the maximum credible population allele frequency of 0.001%,^9^, ^12^ suggests it is unlikely to be a highly penetrant gene with autosomal dominant transmission. This type of critical data, however, was difficult to obtain previously due to prohibitive sequencing costs for thousands of participants without a particular disease phenotype. While large population‐based databases such as the Genome Aggregation Database (gnomAD) are available, they are not ideal for this purpose for two reasons. First, the diabetes phenotype is not available in the public database. Second and more importantly, comparison of mutation carrier rates in studies of diabetes participants with controls from public databases is confounded by several factors such as different sequencing and annotation methods.

An important caveat, however, should be noted when interpreting the results of this indirect approach. A significant difference in mutation carrier rates between cases and controls simply suggests that the mutations are associated with increased risk for diabetes, not specifically for MODY. In fact, considering that approximately 90% of diabetes participants in the general population are type 2 diabetes, the results from this indirect association test are strongly influenced by their risk for type 2 diabetes. Because of this limitation, this approach is not suitable for discovering novel genes specific for MODY. However, for the purpose of confirming known MODY genes, this indirect approach is justified because MODY is a type of diabetes.

It is notable that our findings using statistical method help underscore some of the prior evidence on the causality of MODY candidate genes and question others. Prior evidence has described variable levels of evidence for MODY susceptibility genes^14^, ^15^, ^16^ *HNF1A, HNF4A, GCK, HNF1B, ABCC8, KCNJ11* and *INS* have been thought to be the more common well‐established forms of MODY.^16^, ^17^ Of these well‐established genes, this study found convincing statistical evidence to support *HNF1A, HNF4A, GCK, HNF1B* and *KCNJ11*. We found that *INS* and *ABCC8* were inconclusive: (1) *INS* fulfilled the frequency criteria, but only one P/LP variant was found in a subject without diabetes. A larger sample size may be needed to move this gene into a more definitive consistent or inconsistent category. (2) *ABCC8* was more common in diabetes versus non‐diabetes, but the aggregate allele frequency was higher than the maximum credible population frequency. It is possible that some of classified P/LP mutations are not highly penetrant for diabetes. Our results support recent findings that dispute the MODY causality of *KLF11, PAX4* and *BLK* ^17^, ^18^ which we classified as either inconsistent or inconclusive.

Evaluating diabetes‐related characteristics between carriers and non‐carriers of P/LP mutation provides additional evidence for differentiating the 17 genes. In diabetes participants, typical MODY features (early age of diagnosis, stronger family history and low BMI) were found only in mutation carriers of the ‘consistent’ genes, but not for the other genes. In participants without diabetes diagnosis, significantly higher proportion of family history and higher HbA1C were also found only in mutation carriers of the ‘consistent’ genes, but not for the other genes. The findings of the ‘consistent’ genes in non‐diabetes participants suggest incomplete penetrance of these genes. It also highlights the complexity of MODY and emphasizes that it is not a classic Mendelian dominant disease.

The finding that 10 of the 17 known MODY genes are either inconclusive or inconsistent for being high penetrant for diabetes is surprising and differs from many previous reports.^3^, ^4^, ^5^, ^9^, ^19^, ^20^, ^21^, ^22^, ^23^, ^24^, ^25^, ^26^, ^27^, ^28^ Our results, however, were similar to the population‐based study reported by Flannick and colleagues.^6^ By studying pathogenic nonsynonymous mutations of 7 MODY genes in two large population‐based cohorts, they also found that the mutation carriers did not have elevated risk for diabetes.

Based on a combination of examining data from this current study and reviewing prior evidence, several postulations can be developed to explain the lack of statistical evidence for the 10 inconclusive or inconsistent genes in this study. The first postulation is lack of statistical power in our study to implicate genes with extremely low mutation rates. An example of these genes is *INS* where only one P/LP mutation carrier was found among 200,574 participants in the cohort.

The second postulation is lack of strong prior evidence for some genes. Examples include *BLK*, *PAX4* and *APPL1*.^26^, ^27^, ^28^ These genes were proposed primarily based on observations of several coding mutations in probands and co‐segregation in limited numbers of small pedigrees. Because of limited evidence, these genes have been recently proposed for removal from candidate MODY genes.^2^ Furthermore, non‐expression of *BLK* and *PAX4* in pancreatic tissues suggests they are unlikely MODY‐causal genes.

The third postulation is that several genes are involved in other types of diabetes but do not necessarily cause MODY via haploinsufficiency. Examples for these genes include *WFS1*.^3^ Mutations in *WFS1* are responsible for Wolfram syndrome, a recessive inherited disease.^29^ Common sequence variants in the *WFS1* region were implicated in type 2 diabetes risk from genome‐wide association studies.^30^ In addition, several rare sequence variants in *WFS1* were also associated with type 2 diabetes risk in large exome sequencing studies.^31^ Their risk for type 2 diabetes is modest, with OR at ~1.1 for both common and rare variants. However, evidence for *WFS1*’s role in MODY remains limited. One study reported associations of heterozygous *WFS1* mutations with mild form of Wolfram syndrome and type 2 diabetes in Ashkenazi Jewish individuals.^3^ Another study found a dominant nonsynonymous mutation in *WFS1* co‐segregated with nonsyndromic adult‐onset diabetes in a Finnish family.^32^ Sample sizes were relatively small in both studies. In our study, two pieces of results for *WFS1* in diabetes participants (mutation carrier rate was primarily found in type 2 diabetes participants and carriers had lower proportion of family history and higher BMI compared to non‐carriers) suggest it is more likely associated with risk for type 2 diabetes than MODY. Association of several MODY genes with type 2 diabetes risk was also found in several large cohorts.

Leveraging statistical evidence to identify genes with high penetrance for MODY has clinical implications. Most commonly, clinicians attempt to utilize different criteria (‘atypical diabetes’, normal BMI and early‐onset type 2 diabetes, the MODY probability calculator) to identify people who may have a high probability of MODY.^33^ Results from MODY genetic testing can be confusing as to whether the mutation is causing the clinical features. Clinicians may use resources like ClinVar, genetic testing reports, or monogenic diabetes experts and cohorts to determine whether a variant is MODY‐causing. The clear limitation is that genetic testing is only done in people with these particular features (testing bias) and is not done on a large scale. Large phenotype‐genetic data sets allow clinicians to examine a mutation on a population level without the need to have it associated with a certain MODY phenotype prior to genetic testing.^34^ Ultimately, as whole‐exome sequencing is translated into usual clinical practice, this statistical approach will be crucial in validating whether genes are causal to a disease. This study at least reveals the complexity and suggests caution should be exercised when interpreting germline testing results.

Our study has several limitations. First, despite the large sample size in our study, the statistical power remains limited for genes with extremely rare P/LP mutation carrier rates such as *INS*. Therefore, caution should be taken when interpretating negative results. Second, no MODY diagnosis is available in our study. Furthermore, only limited diabetes‐related phenotypes are available in participants with and without diabetes. This limitation decreases our ability to perform in‐depth phenotype analyses. Third, although we followed the general ACMG and SVI guidelines of these genes, we acknowledged the possibility of misclassification (both under‐ and over‐calling of P/LP mutations) or differences from prior publications.^34^ The reasons for this include (1) the guidelines for classifications are currently under review (Pollin TP, Monogenic Diabetes Expert Panel Variant Curation),^8^, ^35^ (2) the current sequence analysis was based on PLINK data from UKB versus VCF data, and (3) we examined variants within the target region of exome ±2 base pairs of the flanking region. For example, one pathogenic *PDX1* mutation was found by Udler et al., but this was not in our target region. Furthermore, we were unable to evaluate large‐sized germline deletions and gains in this study. Both under‐ and over‐calling mutations may weaken the statistical evidence. Finally, because approximately 95% of study participants in our study are Caucasians, mutations that are unique in minority populations may be overlooked. Similar studies in minority populations are needed.

## 5 CONCLUSIONS

Data from this large population‐based study provide novel statistical evidence for the 17 candidate MODY genes. While 6 were found to be consistent with being highly penetrant for diabetes, the remaining 11 were either inconclusive or inconsistent. These results have potential implications for interpretating genetic testing results and clinical diagnosis of MODY.

## AUTHOR CONTRIBUTIONS

**Liana K. Billings:** Conceptualization (equal); data curation (equal); project administration (equal); writing – original draft (supporting); writing – review and editing (supporting).

## CONFLICT OF INTEREST

Dr. Billings reported receiving advisory/consulting fees from Lilly, Novo Nordisk, Bayer and Sanofi‐Aventis, which are unrelated to this current study. There are no conflicts to report for all other authors.

## ETHICS STATEMENT

The UK Biobank was approved by North West—Haydock Research Ethics Committee (REC reference: 16/NW/0274; IRAS project ID: 200778). Data from the UK Biobank were accessed through a Material Transfer Agreement under Application Reference Number: 50295. This study was performed in accordance with the Declaration of Helsinki.

## Supporting information


**TABLE S1** Manually annotated variants of 17 known MODY genes identified in the UK Biobank
**TABLE S2** Estimated maximum credible population allele frequency for known MODY genes
**TABLE S3** Mutation carrier rates of known MODY genes in participants with and without a diagnosis of diabetes in the UK Biobank
**TABLE S4** Key characteristics of subjects with diabetes diagnosis by mutation carrier status in the UK Biobank

## Data Availability

Datasets relevant to this study are in publicly available repositories (UK Biobank, https://www.ukbiobank.ac.uk/) or included as supplementary material in this manuscript. For questions regarding data availability, please contact the corresponding author, Liana Billings, lbillings@northshore.org.
